# Dental Pulp Stem Cell-Derived Secretome-Induced Reprogramming of Tongue Tumor Microenvironment

**DOI:** 10.1055/s-0046-1816080

**Published:** 2026-02-16

**Authors:** Ferry Sandra, Dewi Ranggaini, Johni Halim, Natalia Tjingson, Melanie Sadono Djamil, Janti Sudiono, Muhammad Ihsan Rizal, Nurrani Mustika Dewi, Alifah Evi Scania, Kyung Hoon Lee

**Affiliations:** 1 Department of Biochemistry and Molecular Biology, Division of Oral Biology, Faculty of Dentistry, Universitas Trisakti, Indonesia; 2 Center of Molecular Biology Study, Faculty of Dentistry, Universitas Trisakti, Jakarta, Indonesia; 3 Department of Physiology, Division of Oral Biology, Faculty of Dentistry, Universitas Trisakti, Jakarta, Indonesia; 4 Master of Dental Science Program, Faculty of Dentistry, Universitas Trisakti, Jakarta, Indonesia; 5 Department of Oral and Maxillofacial Pathology, Faculty of Dentistry, Universitas Trisakti, Jakarta, Indonesia; 6 The Prodia Education and Research Institute, Jakarta, Indonesia; 7 Research Institute, Ballys Co. Ltd, Incheon, Republic of Korea

**Keywords:** dental pulp stem cells, secretome, tumor microenvironment, tongue squamous cell carcinoma, extracellular vesicles

## Abstract

Oral tongue cancer is among the most aggressive malignancies in the head and neck region, driven by a complex tumor microenvironment (TME) that fosters tumor progression, immune evasion, and therapy resistance. Conventional treatments often fail to address the stromal and immunological intricacies of the TME, highlighting the need for microenvironment-targeted therapies. One promising strategy involves the use of dental pulp stem cell-derived secretome (DPSC-Sec), which contains a broad range of bioactive molecules, cytokines, chemokines, growth factors, and extracellular vesicles enriched with regulatory microRNAs. This review explores the potential of DPSC-Sec as a reprogramming agent for modulating the TME in tongue cancer. Evidence suggests that DPSC-Sec may inhibit cancer-associated fibroblast activation, reprogram immunosuppressive cells, remodel the extracellular matrix, normalize aberrant angiogenesis, and regulate oncogenic signaling pathways. The therapeutic quality of DPSC-Sec is significantly influenced by priming methods, which can enhance its potency. While preclinical data are promising, clinical translation requires validation in orthotopic and immunocompetent models, GMP-compliant production, and thorough safety evaluation, especially regarding tumorigenicity and angiogenic effects. Integration with biomaterials, nanocarriers, or conventional therapies may further boost efficacy. This review consolidates current findings on DPSC-Sec and its mechanisms in TME modulation, underscoring its translational potential and future directions for developing targeted therapies in tongue cancer.

## Introduction

The tumor microenvironment (TME) in oral squamous cell carcinoma (OSCC) comprises cancer cells, cancer-associated fibroblasts (CAFs), endothelial and immune cells, extracellular matrix (ECM) components, microbiome, and soluble factors such as cytokines and growth factors. 1 2 Rather than serving as a passive backdrop, the TME actively drives tumor progression through bidirectional communication between malignant and nonmalignant cells. 3 In OSCC, CAFs and immunosuppressive subsets, including tumor-associated macrophages (TAMs) and regulatory T cells (Tregs), establish a permissive niche that fosters tumor growth and shields cancer cells from immune attack. 4

Given this central role, therapeutic strategies increasingly aim to reprogram rather than simply eradicate the TME. Such “microenvironmental reprogramming” seeks to disrupt tumor-promoting signaling, restore antitumor immunity, and potentially redifferentiate malignant cells toward a less aggressive phenotype. 1 5 Unlike conventional cytotoxic therapies, which often cause collateral damage to normal tissue, TME modulation offers a more targeted and potentially less toxic approach. 6 Among the emerging strategies, stem cell-derived bioactive factors have drawn particular attention as versatile modulators of the TME. 7

Within this context, the dental pulp stem cell-derived secretome (DPSC-Sec) has gained interest. It contains anti-inflammatory cytokines and extracellular vesicles (EVs) enriched with regulatory microRNAs that, based on mesenchymal stem cell (MSC) secretome studies, may reduce cancer cell viability, inhibit proliferation, induce apoptosis, and remodel the TME. 8 9 Secretomes derived from bone marrow, adipose tissue, and umbilical cord MSCs have demonstrated both anti- and pro-tumorigenic effects depending on tumor type, experimental conditions, and disease stage, highlighting the importance of tissue origin in determining biological activity. 10 However, secretome activity is highly context-dependent, and findings from non-oral MSC sources cannot be directly extrapolated to oral cancers. 10 In this regard, the neural crest and oral tissue origin of DPSCs may confer a secretory profile with distinct relevance to the oral TME. 11

Despite encouraging insights from regenerative studies, direct evidence of DPSC-Sec effects in tongue cancer remains limited. This gap is clinically relevant given the poor prognosis of advanced oral cancers and the urgent need for microenvironment-targeted strategies. 12 DPSC-Sec offers two potential advantages: its tissue origin may confer specific interactions within the oral TME, and its paracrine factors can act simultaneously on multiple microenvironmental components. 12 By disrupting stromal support, enhancing antitumor immunity, and interfering with pro-survival signaling, DPSC-Sec may reprogram the TME from a tumor-promoting to a tumor-suppressive state.

This review explores the current understanding of DPSC-Sec biology, elucidates its potential mechanisms of action in modulating the tongue TME, and evaluates its prospects for clinical translation.

## TME in Tongue Cancer

Contrary to the traditional view that malignancy is solely driven by intrinsic genetic alterations, it is now clear that the TME exerts an equally decisive influence on initiation, progression, and therapy resistance. 13 This is particularly relevant in tongue squamous cell carcinoma (TSCC), where anatomical and stromal features shape unique tumor–host interactions. 14

A hallmark of the tongue TME is the activation of fibroblasts into CAFs. 15 These cells secrete cytokines such as IL-6 and TGF-β, which promote epithelial–mesenchymal transition (EMT), invasion, and metastasis. 16 Within the muscle-rich stroma of the tongue, CAF-driven deposition of collagen and fibronectin, together with matrix metalloproteinases (MMPs), stiffens the ECM, facilitates migration, and enhances mechanotransduction pathways such as YAP/TAZ. 16 This barrier effect also limits drug penetration and contributes to chemotherapy resistance.

Immune suppression is another defining feature. Tongue cancers recruit Tregs, myeloid-derived suppressor cells (MDSCs), and M2-polarized TAMs, which secrete Interleukin 10 (IL-10) and vascular endothelial growth factor (VEGF) to dampen cytotoxic T cell activity and promote angiogenesis. 13 Compared with other oral subsites, tongue tumors often show higher infiltration of Forkhead Box P3 (FOXP3 ^+^ ) Tregs and M2 TAMs, tipping the balance toward immune evasion and explaining their more aggressive clinical behavior. 17 Meanwhile, cluster of differentiation 8 ^+^ (CD8 ^+^ ) T cells and natural killer cells (NK cells) are functionally impaired, further compromising antitumor immunity. 18 19 The highly vascularized and lymphatic anatomy of the tongue compounds these effects, fueling growth and facilitating early cervical nodal metastasis. 19

These stromal and immune cues converge on key oncogenic signaling pathways. Paracrine growth factors from CAFs and TAMs activate phosphoinositide-3 kinase (PI3K)/protein kinase B (Akt)/mechanistic target of rapamycin (mTOR), mitogen-activated protein kinase (MAPK) **/** extracellular signal–regulated kinase (ERK), 20 and nuclear factor kappa light chain enhancer of activated B cells (NF-κB) signaling, promoting proliferation, survival, and chronic inflammation. 21 Such feedback loops entrench malignancy and help explain why tongue cancers often recur locally and metastasize earlier than other OSCC subsites. 21

Importantly, the TME functions not only as a driver of disease but also as a barrier to effective treatment. Dense stroma hampers drug delivery, and immunosuppressive signaling undermines immunotherapy. 1 This is further complicated by the fact that conventional therapies can remodel the TME in ways that favor persistence, such as enriching resistant cancer stem cell populations. 4 These limitations highlight the need to treat the TME itself as a therapeutic target. In tongue cancer, strategies such as CAF inhibition and TAM reprogramming show promise but remain constrained by off-target effects and limited efficacy. 4 This gap underscores the potential of innovative approaches, such as stem cell-derived secretomes that can act on multiple TME components simultaneously, as shown in Fig. 1 . For clarity, the main TME components in tongue cancer are summarized in Table 1 .

**Table 1:** Major components of the TME

TME component	Key mediators	References
CAFs	IL-6, TGF-β, MMPs, collagen, fibronectin	15
TAMs, M2 phenotype	IL-10, VEGF, growth factors	17
Tregs and MDSCs	IL-10, TGF-β	13
Dysregulated signaling pathways	PI3K/Akt, MAPK/ERK, NF-κB	21
Therapy-induced remodeling	Adaptive stromal responses, cancer stem cell enrichment	4

**Fig. 1:**
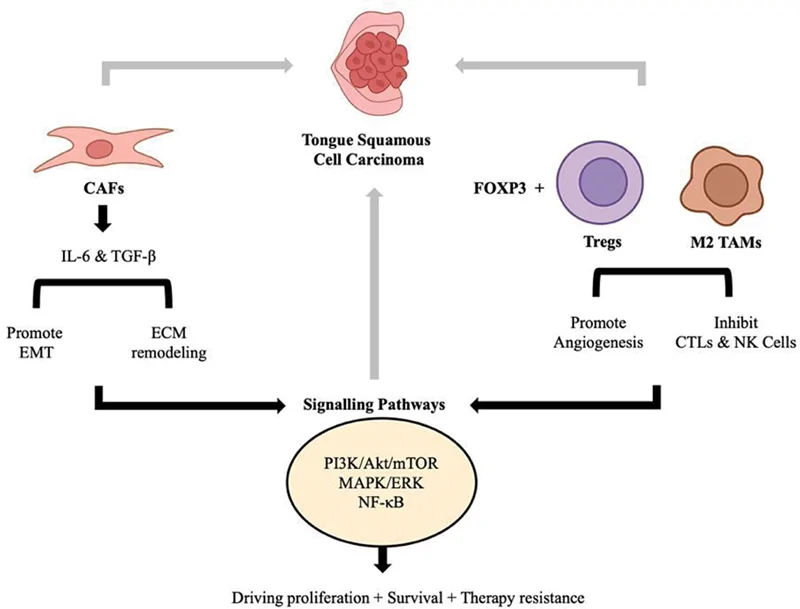
TME in tongue squamous cell carcinoma. CAFs secrete IL-6 and TGF-β to promote EMT, ECM remodeling, and invasion. Immunosuppressive cells such as FOXP3 ^+^ Tregs and M2 TAMs inhibit cytotoxic T lymphocytes (CTLs) and NK cells while enhancing angiogenesis. These stromal and immune interactions converge on PI3K/Akt/mTOR, MAPK/ERK, and NF-κB signaling, driving proliferation, survival, and therapy resistance.

## Dental Pulp Stem Cells

DPSCs are a distinct population of MSCs derived from the dental pulp, the soft connective tissue located in the central cavity of teeth. 22 DPSCs are present in permanent form and originate from neural crest-derived ectomesenchyme, which contributes to the formation of multiple craniofacial tissues, including dental pulp. 23 They are primarily localized in the cell-rich zone adjacent to neurovascular bundles. 24

DPSCs can be harvested from extracted permanent teeth, particularly impacted third molars or premolars removed for orthodontic purposes. 25 DPSCs exhibit classical MSC features, including multipotency, high proliferative activity, and immunomodulatory capacity. 22 They differentiate into odontoblasts, osteoblasts, chondrocytes, adipocytes, neural-like cells, and vascular/endothelial lineages. 26 Their high colony-forming efficiency and rapid proliferation further confirm their regenerative potential. 27

DPSCs modulate immune responses and exert immunosuppressive effects. 28 29 They express typical MSC surface markers, including CD29, CD44, CD73, CD90, CD105, CD146, and Stro-1. 30 31 Compared with other oral-derived stem cells, such as gingival MSCs (GMSCs), stem cells from the apical papilla (SCAP), and stem cells from exfoliated deciduous teeth (SHED), DPSCs are the most extensively studied. 32 Their ease of procurement through minimally invasive procedures such as routine tooth extraction, combined with low ethical concerns, enhances their clinical attractiveness relative to bone marrow-, adipose-, or embryonic-derived stem cells. 33 34

### DPSC Secretome

Beyond their intrinsic properties, DPSCs exert therapeutic effects largely through their DPSC-Sec, defined as the repertoire of bioactive factors they release, including soluble proteins and EVs. 35 The soluble protein fraction contains cytokines, chemokines, and growth factors. 36 37 These regulate angiogenesis, neuroprotection, immune modulation, and tissue regeneration. 38 The EV fraction consists of exosomes and microvesicles carrying proteins, lipids, and nucleic acids such as microRNAs, enabling intercellular communication, gene regulation, and modulation of the TME. 39 Collectively, these components highlight the regenerative and immunomodulatory capacity of DPSC-Sec. Representative examples are summarized in Table 2 .

**Table 2:** Components of DPSC-Sec in general

Component	Examples	References
Soluble proteins		
Cytokines	IL-10, TNF-α, IL-6, IL-8, IL-1β, IL-4, IL-13, IFN-γ	28 35
Chemokines	MCP-1/CCL2, RANTES/CCL5, Eotaxin-3/CCL26, CXCL12 (SDF-1), CXCL8, CXCL10	33 38
Growth factors	HGF, TGF-β3, VEGF-A, VEGF-C, NGF, BDNF, FGF-2, PDGF-BB, IGF-1, EGF	22 39
Proteases	MMP-2, MMP-3, MMP-9, MMP-13, TIMP-1, TIMP-2	27
Extracellular vesicles		
Exosomes	- Proteins: HSP70, Alix, TSG101, CD9, CD63, CD81 - Lipids: sphingomyelin, phosphatidylserine, ceramide, cholesterol - Nucleic acids: miR-21, miR-126, miR-146a, miR-34a, miR-210, mRNAs for angiogenic/neurogenic factors	38 39
Microvesicles	Integrins (α4β1, α6β4), annexins (Annexin II, Annexin V), phosphatidylcholine, cholesterol, mRNAs for VEGF, HGF, and osteogenic factors	26

## Preclinical Evidence of DPSC-Sec In Oral Cancer Models

While the molecular composition of dental pulp stem cell–derived secretome (DPSC-Sec) has been increasingly characterized, its biological effects in cancer have been primarily explored through preclinical in vitro and in vivo models. This section summarizes the current experimental evidence evaluating DPSC-Sec across different cancer settings, with particular attention to OSCC and TSCC, and highlights the context-dependent nature of its effects.

### In Vitro Studies

Most experimental evidence on DPSC-derived secretome (DPSC-Sec) in oncology originates from in vitro studies employing established cancer cell lines. As summarized in Table 3 , these investigations predominantly used DPSC-conditioned medium (CM) and evaluated its effects across OSCC models, including CAL27, HSC-3, SCC-9, and SCC-25, as well as non-oral malignancies such as breast cancer (MCF-7), melanoma (A375), and colorectal cancer (HT-29). 7 40 41 42

**Table 3:** In vitro studies evaluating the effects of DPSC-derived secretome in cancer models

Cancer type	Cell line(s)	DPSC-Sec formulation	Experimental exposure	Main biological effects	Key mechanistic findings	Reference
Oral squamous cell carcinoma (OSCC)	CAL27	Conditioned medium (CM)	24–72 h	↓ Cell viability, ↓ proliferation, ↑ apoptosis	Modulation of PI3K/Akt signaling, caspase activation	7
	HSC-3	Conditioned medium (CM)	48 h	↓ Proliferation, ↓ migration	Interference with EMT-associated phenotypes	40
	SCC-9, SCC-25	Conditioned medium (CM)	24–48 h	↑ Apoptosis, ↓ clonogenic survival	Caspase-dependent apoptotic pathways	40
	Not specified	Conditioned medium (CM)	24–72 h	↑ Proliferation (pro-tumorigenic effect)	Enrichment of growth factors and cytokines	41
Breast cancer	MCF-7	Conditioned medium (CM)	48 h	↓ Proliferation, ↑ apoptosis	MAPK/ERK pathway modulation	7
Melanoma	A375	Conditioned medium (CM)	48 h	↓ Migration and invasion	Altered cytoskeletal and EMT-related signaling	7
Colorectal cancer	HT-29	Conditioned medium (CM)	48–72 h	↓ Proliferation, ↑ apoptosis	Suppression of MAPK signaling cascades	62

Across multiple OSCC cell lines, exposure to DPSC-CM for 24 to 72 hours was most frequently associated with reduced cell viability and proliferation, induction of apoptosis, and suppression of migratory or clonogenic capacity. 7 40 These biological effects were mechanistically linked to modulation of oncogenic signaling pathways, including PI3K/Akt and MAPK/ERK cascades, activation of caspase-dependent apoptotic pathways, and interference with EMT associated phenotypes. 7 ˒ 42 Similar antiproliferative and proapoptotic responses were also observed in nonoral cancer models, indicating that DPSC-derived paracrine factors can exert broad bioactivity across different tumor types. 7 41

However, in vitro findings are not uniformly antitumorigenic. One study demonstrated that DPSC-derived conditioned medium enriched in growth factors and cytokines promoted oral cancer cell proliferation, highlighting a potential protumorigenic effect under specific experimental conditions. 42 Importantly, this divergence cannot be attributed solely to cancer type, but rather reflects differences in secretome composition, culture conditions, exposure duration, and the intrinsic biological characteristics of the target cell lines, as evident from the heterogeneous experimental designs summarized in Table 3 .

Emerging evidence further indicates anti- or protumorigenic behavior of MSC-derived secretomes is strongly influenced by secretome priming strategies, which critically shape the molecular cargo of soluble factors and EVs, particularly microRNAs. Priming conditions such as hypoxia, inflammatory cytokines (e.g., IFN-γ, TNF-α), oxidative stress, or pharmacological stimulation have been shown to substantially alter the EV-associated microRNA repertoire, thereby modulating epigenetic regulation within the TME. 43 44 45 Through horizontal transfer of regulatory microRNAs, primed MSC-derived EVs can induce epigenetic reprogramming of cancer cells, CAFs, and immune components, resulting in either suppression of oncogenic signaling and restoration of antitumor immunity, or conversely, reinforcement of tumor-supportive pathways depending on the priming context. 44 46 47

In this regard, microRNAs delivered by MSC-derived EVs are increasingly recognized as key mediators of TME remodeling through posttranscriptional and epigenetic mechanisms, including regulation of EMT, angiogenesis, immune evasion, and therapy resistance. 46 48 Although direct evidence for epigenetic reprogramming mediated by DPSC-Sec–derived EV–microRNAs in TSCC remains limited, analogous findings from other MSC sources strongly support the concept that controlled priming of DPSC secretomes could determine whether their biological effects shift toward tumor-suppressive reprogramming or unintended tumor promotion. 45 47

Collectively, in vitro studies indicate that DPSC-Sec can modulate multiple cancer-related processes, including proliferation, apoptosis, migration, and clonogenic survival. At the same time, these data underscore the necessity for careful optimization and characterization of secretome formulations, as variations in priming conditions and EV microRNA composition may decisively influence epigenetic regulation of the TME, ultimately determining therapeutic efficacy or risk. A comparative overview of these in vitro findings is provided in Table 3 .

### In Vivo Studies

Compared with the extensive in vitro literature, in vivo evidence directly evaluating dental pulp stem cell–derived secretome (DPSC-Sec) in cancer remains very limited. To date, most in vivo studies relevant to oral cancer have not investigated DPSC-Sec specifically, but rather secretomes or EVs derived from mesenchymal stem cells (MSCs) of other oral sources, such as gingiva-derived MSCs or stem cells from human exfoliated deciduous teeth (SHED), as well as from non-oral tissues including adipose tissue, bone marrow, and menstrual blood. 49 50 51 52 53 54 55 56

As summarized in Table 4 , the biological outcomes of MSC-derived secretomes in in vivo oral cancer models span both antitumorigenic and protumorigenic effects. This heterogeneity cannot be attributed solely to cancer type, but instead reflects the combined influence of multiple experimental variables, including MSC source, the nature of the secreted product (e.g., conditioned medium versus EVs), preparation and enrichment methods, dosage, route of administration, and the local TME in which the secretome is applied. 49 50 51 52 53 54 55 56 Thus, divergent outcomes across studies are more appropriately interpreted as context-dependent responses arising from differences in secretome composition and delivery rather than intrinsic properties of oral cancer models alone.

**Table 4:** In vivo studies investigating the effects of mesenchymal stem cell–derived secretomes or extracellular vesicles in oral squamous cell carcinoma models

MSC source	Cancer model	Animal model	Tumor site	Route of administration	Product	Main in vivo outcome	Effect category	Ref.
SHED (stem cells from human exfoliated deciduous teeth)	OSCC (CAL27)	Female BALB/c nude mice	Subcutaneous	Intratumoral	Exosomes (30–150 nm; miR-100–5p, miR-1246)	↓ Tumor volume; ↓ angiogenesis	Anti-tumorigenic	51
Gingiva-derived MSCs (IFN-β–expressing)	TSCC	Male BALB/c nude mice	Subcutaneous	Intratumoral	Conditioned medium	↓ Tumor growth; ↓ angiogenesis	Anti-tumorigenic	50
Gingiva-derived MSCs (GMSCs)	OSCC (CAL27)	Male BALB/c nude mice	Subcutaneous	Intratumoral	Conditioned medium	↓ Tumor volume; ↑ apoptosis; ↓ proliferation	Anti-tumorigenic	52
Adipose-derived MSCs (ADSCs)	OSCC (CAL27)	Female BALB/c nude mice	Subcutaneous	Intratumoral	Conditioned medium	↑ Tumor growth; ↑ proliferation and migration	Pro-tumorigenic	53
Bone marrow MSCs (BMSCs)	OSCC; TSCC	Male BALB/c nude mice	Subcutaneous; tongue (orthotopic)	Tumor only	MSC secretome	↑ Tumor growth; ↑ angiogenesis; ↑ invasion and metastasis; ↓ epithelial integrity	Pro-tumorigenic	54 55
Gingiva-derived MSCs	OSCC (CAL27)	Female BALB/c nude mice	Thigh root	Tumor only	MSC secretome	↑ Tumor growth; ↑ invasion; ↓ apoptosis	Pro-tumorigenic	56
Menstrual blood-derived MSCs (MenSCs)	DMBA-induced oral cancer	Female Syrian golden hamsters	Buccal pouch	Local application	Conditioned medium	↓ Proliferation; ↓ angiogenesis	Anti-tumorigenic	57

Several studies employing secretomes or exosomes derived from oral MSC sources reported antitumorigenic effects, such as reduced tumor volume, suppressed angiogenesis, increased apoptotic signaling, and decreased proliferative indices in OSCC and TSCC xenograft models established in BALB/c nude mice. 49 50 51 52 53 54 55 56 In contrast, secretomes derived from non-oral MSC sources, particularly adipose-derived MSCs and bone marrow–derived MSCs, have been shown to promote tumor growth, angiogenesis, invasion, and metastatic dissemination, while concomitantly reducing epithelial integrity in in vivo OSCC and TSCC models. 52 53 54 55 These opposing findings underscore that the biological activity of MSC-derived secretomes is strongly influenced by the source and composition of the secreted product, rather than by cancer type alone.

Notably, the majority of available in vivo studies rely on subcutaneous or ectopic xenograft models in immunodeficient animals. Such systems do not fully recapitulate the complexity of the tongue TME, which is characterized by dense musculature, extensive vascular and lymphatic networks, and dynamic immune–stromal interactions. Moreover, the predominant use of immunodeficient hosts precludes meaningful evaluation of immune-mediated mechanisms, which are central to proposed TME–modulating effects of MSC-derived secretomes. 50 51 52

To date, direct in vivo evaluation of DPSC-Sec in orthotopic tongue cancer models remains largely absent, representing a critical translational gap. While existing studies using MSC-derived secretomes from other tissue sources provide important contextual insights regarding biological outcomes, routes of administration, and safety considerations, these findings cannot be directly extrapolated to DPSC-Sec or to TSCC. Accordingly, rigorously designed in vivo studies employing orthotopic, immunocompetent models are required to clarify the role of DPSC-Sec in modulating the tongue TME and to support its potential clinical translation.

## Mechanisms of Microenvironment Reprogramming by DPSC-Sec

The DPSC secretome, a complex mixture of EVs, cytokines, growth factors, and microRNAs, offers multiple mechanisms for reprogramming the TME in tongue cancer. 42 43 It can disrupt stromal support, enhance immune surveillance, normalize ECM architecture, modulate angiogenesis, and influence survival and epigenetic pathways in malignant cells. 40 57 The major mediators implicated in each of these TME targets are summarized in Table 5 .

**Table 5:** DPSCs mediator in each target of TME

Target	DPSC-Sec mediators	References
CAFs	miR-146a, HGF, EVs containing anti-fibrotic factors	58
Immune cells (TAMs, Tregs, MDSCs, T cells)	EV-miRNAs, PGE _2_ , IDO	59
ECM	Reduced MMPs, TIMP secretion, regulatory cytokines	59
Angiogenesis	VEGF, HGF, angiogenic miRNAs	59
Key signaling pathways	miR-21 inhibitors, PI3K/Akt regulators, MAPK modulators	59
Epigenetic reprogramming	EV-carried miRNAs, lncRNAs	57

A key feature of DPSC-Sec is its capacity to regulate CAFs, which normally secrete transforming growth factor-β (TGF-β), interleukin 6 (IL-6), and matrix-degrading enzymes that drive invasion, ECM remodeling, and therapy resistance. 58 Bioactive factors within the secretome inhibit CAF activation by blocking TGF-β/Smad signaling, reducing MMP expression, and promoting a quiescent phenotype. This reversal of pro-tumor stroma reduces both physical and paracrine barriers to drug delivery. 58

The immunoregulatory effects of DPSC-Sec are also notable. MSC secretomes can shift macrophages from M2-like toward M1-like polarization, enhancing phagocytic and antitumor activity. 40 59 In addition, prostaglandin E _2_ (PGE _2_ ), indoleamine 2,3-dioxygenase (IDO), and immunoregulatory miRNAs can suppress regulatory T cells and MDSCs while reactivating cytotoxic T lymphocytes and natural killer cells. Given its EV- and miRNA-rich profile, DPSC-Sec may help restore antitumor immunity within the immunosuppressive niche typical of OSCC and TSCC. 60

Beyond stromal and immune targets, DPSC-Sec influences ECM remodeling and angiogenesis. 61 By reducing MMP activity, it can hinder invasion and metastasis. At the same time, factors such as VEGF and HGF may promote angiogenesis but also normalize vasculature, improving immune and therapeutic delivery. 61 Key signaling cascades, including MAPK, PI3K/Akt, and apoptosis pathways, are susceptible to regulation by secretome components such as IGF, TGF-β inhibitors, and regulatory miRNAs, which can suppress proliferation and sensitize cells to apoptosis. 60

Importantly, growing evidence indicates that EV-associated microRNAs can mediate epigenetic and transcriptional reprogramming in TSCC. Studies using TSCC-derived models have demonstrated that tumor- and stroma-derived EVs deliver microRNAs capable of modulating DNA methylation patterns, histone modification–associated regulators, and EMT-related transcription factors, thereby influencing tumor invasiveness, stemness, and therapy resistane. 52 62 For example, EV-carried miRNAs such as miR-21, miR-155, and miR-200 family members have been implicated in epigenetic control of EMT and immune evasion in TSCC through regulation of downstream targets involved in chromatin remodeling and transcriptional repression. 52 62

Although direct evidence demonstrating EV microRNA-mediated epigenetic reprogramming by DPSC-Sec in TSCC is currently lacking, the established role of EV-miRNA signaling in TSCC provides a strong mechanistic rationale. Given that DPSC-Sec is enriched in regulatory microRNAs and EVs with demonstrated epigenetic activity in other cancer models, 57 it is plausible that similar epigenetic mechanisms may contribute to its TME-modulating effects in tongue cancer. This hypothesis warrants direct validation in orthotopic TSCC models and epigenomic profiling studies. Together, these findings position DPSC-Sec as a multitarget modulator of the tongue TME, with potential to reprogram stromal fibroblasts, reshape immune responses, remodel ECM and vasculature, disrupt oncogenic signaling, and, based on emerging EV-miRNA literature, possibly influence epigenetic states that govern malignant cell behavior.

## Methods for DPSC-Sec Isolation and Characterization

Isolation and characterization of DPSC-Sec largely follow standardized protocols designed to ensure purity and functional reliability. Most studies employ serum-free conditioned medium (CM) collection. 35 Typically, DPSCs are cultured to 60 to 80% confluency, washed two to three times with phosphate-buffered saline (PBS), and switched to serum-free or low-serum medium to avoid contamination from serum proteins and vesicles. 63 Cells are then incubated for 24 to 48 hours to allow accumulation of secreted factors. The CM is collected, filtered to remove cells and debris, concentrated (e.g., ∼40-fold) using centrifugal filter units or ultrafiltration, and supplemented with protease inhibitors to prevent degradation. 64 In some cases, metabolic or isotopic labeling strategies are applied to increase specificity for truly secreted proteins. 65

Although most studies analyze total CM, DPSC-Sec can also be enriched for EVs. The most common method is ultracentrifugation, in which sequential spins remove debris, followed by pelleting of small EVs at ∼100,000 ×g. 66 For higher purity, pellets can be processed using iodixanol density gradients or size-exclusion chromatography, separating EVs from protein aggregates and lipoproteins. 67 Large-scale preparations may also use filtration or precipitation approaches. Isolated EVs should be stored at 4 °C for ≤48 hours or at −80 °C in sterile PBS (optionally with cryoprotectant), while repeated freeze–thaw cycles should be avoided. 67

In addition to cargo analysis, basic EV characterization is essential to validate vesicle identity and quality in accordance with current MISEV guidelines. EV size distribution and particle concentration are commonly assessed using nanoparticle tracking analysis (NTA), which typically reveals vesicles in the 30- to 150-nm range for small EVs. 68 Morphological confirmation is performed using transmission electron microscopy (TEM) or cryoelectron microscopy, demonstrating the characteristic cup-shaped or spherical lipid bilayer structures. 69 Surface and intravesicular markers are evaluated by Western blotting or flow cytometry, with positive markers including tetraspanins (CD9, CD63, CD81), TSG101, and Alix, while negative markers such as calnexin or GM130 are used to exclude contamination from intracellular organelles. 68 70 This multiparametric characterization ensures the purity, reproducibility, and biological relevance of DPSC-derived EV preparations.

A wide range of analytical methods is used to characterize the secretome. Proteomic approaches such as liquid chromatography–tandem mass spectrometry (LC-MS/MS) allow comprehensive protein profiling, 71 while cytokine arrays and ELISA enable targeted quantification of immunoregulatory factors. 63 High-performance liquid chromatography (HPLC) can be used to validate and quantify proteins such as bone morphogenetic proteins. Functional assays confirm biological activity in processes like angiogenesis, neuronal survival, and immune modulation. 72 EVs within the secretome are further characterized using NTA, TEM, and Western blotting for vesicle-specific markers. 71 These isolation and analytical strategies, summarized in Table 6 , provide a comprehensive molecular and functional profile of DPSC-Sec, supporting its application in regenerative medicine and tissue engineering.

**Table 6:** DPSC-Sec characterization techniques

Technique	Purpose	Example application in DPSC studies	Ref.
LC-MS/MS proteomics	Identify and quantify secreted proteins	Comprehensive secretome profiling	71
Cytokine arrays/ELISA	Quantify cytokines and/or growth factors	Compare DPSC-Sec to other MSCs	34
HPLC	Protein separation and quantification	BMP-2 detection in DPSC secretome	72
Functional assays	Assess biological activity	Anti-inflammatory, neuroprotective effects	64

## Priming of Dental Pulp Stem Cells

Priming refers to the process of preconditioning stem cells to activate their functional potential before therapeutic application. In other words, stem cells can work optimally only after being primed. 73 This concept is particularly relevant for DPSCs, whose secretory activity can be significantly enhanced through specific priming strategies. By stimulating DPSCs with defined environmental cues, their secretome becomes enriched with bioactive molecules such as cytokines, chemokines, growth factors, and EVs, thereby tailoring their biological effects to match therapeutic goals. 74

One of the most widely used methods is inflammatory priming. Exposure to cytokines such as interferon-γ, tumor necrosis factor-α, or interleukin-1β induces a shift toward an immunoregulatory phenotype. This results in the upregulation of key mediators, including IDO, prostaglandin E _2_ , and IL-10, which collectively enhance the anti-inflammatory and immunosuppressive properties of the DPSC secretome. 73 75 By strengthening their capacity to suppress activated immune cells and promote macrophage polarization, inflammatory priming generates secretomes particularly suited for controlling pathological inflammation. 73

Growth factor priming represents another important approach. For example, fibroblast growth factor-2 (FGF-2) has been shown to activate MAPK/PI3K pathways in DPSCs, expand progenitor subpopulations, and markedly increase secretion of proangiogenic factors such as VEGF and HGF. When transplanted, FGF-2-primed DPSCs induce denser vascular networks compared with unprimed cells, underscoring the translational relevance of a secretome enriched in angiogenic signals. 76

Beyond soluble factors, culture conditions themselves can act as priming stimuli. Three-dimensional (3D) spheroid culture has been reported to enhance cell–cell and cell–matrix interactions, thereby altering signaling pathways and gene expression in DPSCs. 77 Compared with conventional monolayer cultures, 3D spheroids secrete greater amounts of angiogenic cytokines such as VEGF and anti-inflammatory mediators such as IL-1Ra. This priming method also preserves stemness, enhances viability, and results in a secretome with improved trophic and immunomodulatory potential. 78

In vivo priming offers another level of complexity by conditioning DPSCs within disease-relevant environments. 76 Implantation into host tissues exposes them to a combination of biological signals that modify their secretory profile. Studies have shown that primed DPSCs transplanted into models of pulp injury or engineered scaffolds produce secretomes enriched with angiogenic and odontogenic factors, supporting tissue vascularization and pulp-like tissue formation. 76 This strategy enhances translational relevance, as the harvested secretome reflects in situ biological signaling.

Finally, tumor-conditioned priming illustrates how DPSCs can respond to pathological microenvironments. Co-culture with cancer cells, exposure to tumor lysates, or treatment with tumor-derived exosomes may alter the DPSC secretome in ways that affect tumor-associated pathways. While some evidence indicates that DPSC-conditioned medium can inhibit proliferation and induce apoptosis in cancer cells through modulation of MAPK/ERK or Akt signaling, 41 caution remains necessary because tumor-derived cues could also favor immune evasion. Nevertheless, tumor-conditioned priming provides a model for exploring how pathological environments can be harnessed or redirected for therapeutic purposes. The main findings of these priming approaches are summarized in Table 7 .

**Table 7:** Biological effects of primed stem cell secretome

Priming strategy	Stimuli/Conditions	Mechanistic changes in stem cells	Biological effects of primed secretome	Ref.
Inflammatory priming	Cytokines (IFN-γ, TNF-α, IL-1β) in culture	↑ Immunomodulatory pathways (e.g., STAT1/NF-κB activation), upregulation of IDO, PGE _2_ , IL-10, chemokine receptors	Enhanced anti-inflammatory/immunosuppressive secretome; potent inhibition of activated immune cells	73
Growth factor priming	FGF-2 supplementation	Activation of MAPK/PI3K signaling; ↑ Stro-1 ^+^ /CD146 ^+^ progenitors; ↑ expression of VEGF, HGF	Enriched pro-angiogenic secretome; promotes endothelial proliferation and vascularization in grafts	76
3D spheroid culture	Scaffold-free 3D aggregation (spheroids)	Enhanced cell–cell/ECM signaling; ↑ MAPK & NF-κB pathways; upregulation of osteogenic/angiogenic genes	Secretome with increased trophic factors (e.g., IL-1Ra, VEGF); improved multipotency and angiogenic support	77 78
In vivo priming	Transplantation in animal/disease models	Exposure to in vivo cues (growth factors, immune signals); modulation of PI3K-Akt, MAPK pathways	Enhanced tissue regeneration factors – e.g., greater odontogenic and angiogenic factor release, leading to superior pulp formation and neovascularization	76
Tumor-conditioned priming	Co-culture or treatment with tumor cell CM/exosomes	Activation of tumor-influenced signaling (e.g., MAPK/Akt); altered cytokine profile	Alters secretome toward anti-tumor activity (e.g., inhibiting cancer cell proliferation and inflammation via MAPK/Akt modulation)	41

## Translational Perspectives and Future Directions

The translation of DPSC-derived secretomes into therapeutic strategies for tongue cancer depends on optimizing delivery of their paracrine cargo, integrating them with standard-of-care treatments, and addressing safety and manufacturing challenges. 79 Therapeutic delivery strategies can be broadly grouped into direct application, biomaterial scaffolds, and engineered nanocarriers or EVs. Direct intratumoral or peritumoral administration is feasible for oral lesions but is limited by the protease-rich milieu that reduces cargo stability. 79 Biomaterials such as injectable or thermoresponsive hydrogels improve local retention and release kinetics, as demonstrated in immunotherapy models, supporting their relevance for oral cancers. 60 Engineered nanoparticle and EV platforms developed through electroporation, sonication, or membrane fusion enable systemic delivery with targeted tropism, stabilizing the diverse molecular components of DPSC secretomes. 80 These approaches and their translational considerations are summarized in Table 8 .

**Table 8:** Delivery strategies and translational considerations for DPSC-secretomes in tongue cancer

Approach	Advantages	Limitations in oral cancer context	Supporting evidence
Direct/local injection (intratumoral or peritumoral)	Simple, feasible for accessible lesions; high local concentration	Rapid clearance; protease-rich TME degrades factors; limited systemic reach	MSC-EVs in local delivery showed efficacy in solid tumors 60
Hydrogel scaffolds (injectable, thermoresponsive)	Prolonged local retention; tunable release kinetics; can co-deliver drugs	Biodegradation rate must match release needs; risk of immune reaction to scaffold	Hydrogels improved EV retention in tumor models 60 79
Nanocarriers/engineered EVs (electroporation, sonication, fusion)	Systemic delivery; targeted tropism; stabilize heterogeneous cargo	Manufacturing complexity; biodistribution must be tightly controlled	EV-based nanocarriers used for chemo- and immunotherapy delivery 80

**Table 9:** Future research priorities for DPSC-secretome translation in tongue cancer

Research priority	Rationale	Key considerations	Supporting evidence
Orthotopic, of tongue cancer models	Mimic human TME and immune interactions	Assess dosing, schedule, immune modulation	Gap identified in current DPSC studies 7
Biomarker discovery & validation	Predict responders; monitor treatment response	EV-miRNAs (miR-21, let-7a, miR-181a) in HNSCC serum	HNSCC biomarker studies 88 89 90
Safety and GMP production standards	Prevent tumorigenic or pro-angiogenic effects; regulatory compliance	Evaluate off-target effects, procoagulant activity	Regulatory warnings on unapproved exosome use 88 90
Combination strategies with SOC (chemo/radio/ICI)	Exploit synergy with immune checkpoint blockade or cytotoxic therapies	Optimize schedules to avoid antagonism	EVs as immunotherapy enhancers 76 87

Beyond delivery, scalability of secretome production represents a major bottleneck for clinical translation. Most experimental studies still rely on small-scale, manually operated two-dimensional (2D) culture systems, such as tissue culture flasks, which suffer from limited yield, batch-to-batch variability, and poor reproducibility. Transition toward scalable manufacturing platforms, including multilayer flasks, hollow-fiber bioreactors, stirred-tank bioreactors, and microcarrier-based dynamic culture systems, has been proposed to enable large-scale production of Good Manufacturing Practice-compliant MSC secretomes and EVs. These bioreactor-based systems allow precise control of oxygen tension, shear stress, nutrient supply, and priming conditions, which are critical determinants of secretome composition and bioactivity. 81 82 83 Importantly, dynamic culture environments have been shown to enhance EV yield and modulate microRNA cargo compared with conventional static cultures, underscoring their relevance not only for scalability but also for functional consistency. 82 84

Given the complexity of the TME, combinatorial approaches are rational. DPSC-Sec can normalize vasculature, suppress CAF signaling, and restore antitumor immunity, thereby enhancing responsiveness to chemotherapy, radiotherapy, or immune checkpoint blockade. 85 EVs are increasingly used as delivery vehicles for immunomodulatory agents, with engineered EVs carrying tumor antigens showing synergy with PD-(L)1 and CTLA-4 inhibitors. 85 86 Biomarker-based studies in head and neck squamous cell carcinoma (HNSCC) also suggest that secretome-mediated remodeling of the tumor immune microenvironment (TIME) may sensitize tumors to checkpoint inhibitors. 87

Biomarker-guided patient stratification represents a critical translational requirement. Candidate biomarkers for DPSC-Sec responsiveness include changes in immune-related parameters such as CD8 ^+^ T cell infiltration, PD-L1 expression, macrophage polarization markers, and soluble cytokines including IL-6, TGF-β, and IFN-γ. In addition, EV-associated microRNAs such as miR-21, let-7a, and miR-181a have emerged as potential prognostic and predictive biomarkers in HNSCC. 88 89 90 Validation of these biomarkers requires integrated approaches combining high-throughput omics analyses, orthotopic in vivo models, and longitudinal assessment in patient-derived samples.

Safety remains a key concern. MSC secretomes show context-dependent effects and may, in some cases, promote angiogenesis or tumor progression. 91 Regulatory agencies caution that unapproved “exosome” therapies pose risks, classifying EVs as biological products requiring GMP standards and rigorous safety testing. 92 Although DPSC secretomes have shown low immunogenicity in regenerative contexts, oncology applications require evaluation of tumorigenic potential, biodistribution, and procoagulant activity. 93 From a manufacturing perspective, major challenges include scalable production of GMP-grade secretomes, standardization of isolation and storage protocols, quality control of bioactive cargo, and long-term stability.

Establishing closed, automated bioreactor-based workflows integrated with standardized downstream purification and release criteria is therefore essential to meet regulatory expectations and ensure clinical reproducibility. Addressing these issues is essential to ensure reproducibility and regulatory approval for clinical trials ( Table 9 ).

## Conclusion

TME plays a pivotal role in tongue cancer progression and therapeutic response. DPSC-Sec shows potential to reprogram the TME by modulating fibroblasts, immune responses, vasculature, ECM, and regulatory RNAs. Current evidence suggests cytostatic and immunomodulatory effects, though outcomes may vary depending on context and preparation. Advancing DPSC-Sec into clinical application requires rigorous in vivo validation, optimized priming and engineering, and standardized manufacturing to ensure safety and efficacy. Tailored strategies, rather than a one-size-fits-all approach, will be essential for leveraging DPSC-Sec in future TME-targeted therapies.
